# In Vivo Imaging of Rat Vascularity with FDG-Labeled Erythrocytes

**DOI:** 10.3390/ph15030292

**Published:** 2022-02-27

**Authors:** Shaowei Wang, Mikalai Budzevich, Mahmoud A. Abdalah, Yoganand Balagurunathan, Jung W. Choi

**Affiliations:** 1 Department of Medical Engineering, University of South Florida, Tampa, FL 33620, USA; shaowei.wang@usf.edu; 2 Small Animal Imaging Laboratory, Department of Cancer Physiology, H. Lee Moffitt Cancer Center and Research Institute, Tampa, FL 33612, USA; mikalai.budzevich@moffitt.org; 3 Department of Diagnostic Imaging and Interventional Radiology, H. Lee Moffitt Cancer Center and Research Institute, Tampa, FL 33612, USA; mahmoud.abdalah@moffitt.org; 4 Division of Machine Learning, Department of Cancer Imaging and Metabolism, H. Lee Moffitt Cancer Center and Research Institute, Tampa, FL 33612, USA; yoganand.balagurunathan@moffitt.org

**Keywords:** vascular imaging, FDG, PET/CT, microvasculature imaging

## Abstract

Microvascular disease is frequently found in major pathologies affecting vital organs, such as the brain, heart, and kidneys. While imaging modalities, such as ultrasound, computed tomography, single photon emission computed tomography, and magnetic resonance imaging, are widely used to visualize vascular abnormalities, the ability to non-invasively assess an organ’s total vasculature, including microvasculature, is often limited or cumbersome. Previously, we have demonstrated proof of concept that non-invasive imaging of the total mouse vasculature can be achieved with 18F-fluorodeoxyglucose (18F-FDG)-labeled human erythrocytes and positron emission tomography/computerized tomography (PET/CT). In this work, we demonstrate that changes in the total vascular volume of the brain and left ventricular myocardium of normal rats can be seen after pharmacological vasodilation using 18F-FDG-labeled rat red blood cells (FDG RBCs) and microPET/CT imaging. FDG RBC PET imaging was also used to approximate the location of myocardial injury in a surgical myocardial infarction rat model. Finally, we show that FDG RBC PET imaging can detect relative differences in the degree of drug-induced intra-myocardial vasodilation between diabetic rats and normal controls. This FDG-labeled RBC PET imaging technique may thus be useful for assessing microvascular disease pathologies and characterizing pharmacological responses in the vascular bed of interest.

## 1. Introduction

Microvascular disease (MVD) is a type of vascular disorder affecting the arterioles, venules, and capillaries of the vasculature [1,2]. Moreover, MVD can be found in vital organs, such as the brain, heart, and kidneys, in various diseases, including atherosclerosis, dementia, and stroke [3,4,5,6,7]. Unfortunately, non-invasive MVD-specific detection methods are limited, making the early diagnosis, characterization, and treatment of MVD challenging. For example, in the heart, coronary microvascular dysfunction (CVD) can occur with or without obstructive epicardial coronary artery disease, leading to ischemia and angina [8,9,10,11]. Imaging techniques, such as coronary flow reserve (CFR), fractional flow reserve (FFR), and index of microcirculatory resistance (IMR), are accepted as measures of myocardial blood flow and even microvascular disease; but are essentially limited to invasive coronary angiography [12,13,14]. Ultrasound microbubble imaging has been used to assess blood flow and velocity in multiple organs but is dependent on adequate acoustic window access to vessels of appropriate size and proximity and is limited by inherent physical microbubble properties [15,16,17]. Measures of blood volume and blood flow in organs using iodine and gadolinium-based extracellular contrast agents in computed tomography (CT) or magnetic resonance imaging (MRI) are either semi-quantitatively performed or require compartmental kinetic modeling involving a sufficiently sized vessel in close proximity to the vascular bed of interest [18,19]. The intravascular MRI imaging agent gadofosveset (Vasovist^®^/Ablavar^®^) had been successfully used to image the whole organ vasculature in patients but was later withdrawn from the market and is no longer available for commercial use [20]. Arterial spin label MRI has also been successfully used to quantify cerebral blood flow but is technically challenging to perform and is sensitive to differences in MRI technique and parameter settings [21]. As a non-invasive imaging technology with high tracer sensitivity, positron emission tomography (PET) has been shown to accurately provide quantitative measurements of total myocardial perfusion using PET perfusion tracers ^82^Rb, ^13^N-NH_3_, and ^15^O-H_2_O [22,23]. Unfortunately, these PET imaging agents currently have minimal clinical implementation due to their restricted applicability owing either to very short tracer half-lives (^13^N, ^15^O) or requiring access from vendor-supplied tracer generators (^82^Rb) [24,25,26]. More recently, the PET perfusion agent F18-flurpiridaz has recently shown much promise in a phase III clinical trial but currently remains limited to myocardial perfusion imaging [27]. As such, facile methods for robust, non-invasive assessment of the total vascularity of an organ of interest are lacking. The availability of such methods would be expected to both improve the clinical diagnosis and management of patients with MVD, as well as facilitate the development of pharmaceutical agents targeting MVD.

We have previously shown that human red blood cells (RBCs) rapidly incorporate the inexpensive and widely available PET tracer 18F-fluorodeoxyglucose (FDG) due to the inherently dense cell surface expression of glucose transporters, resulting in “trapped” FDG metabolites in human RBCs [28]. We then used FDG-labeled human RBCs as an intravascular PET imaging agent to visualize the entire body vasculature of an immunodeficient mouse model with a microPET/CT scanner. Given the high tracer detection sensitivity of PET imaging relative to contrast agents used in other readily available clinical imaging modalities, we sought to determine whether FDG-labeled RBCs can be used to non-invasively detect changes in the total vascular volume, including the microvasculature, of an organ of interest in rats. As proof of concept, we evaluate the ability of FDG RBC PET imaging to characterize the total vasculature of the brain and left ventricular (LV) myocardium of rats under both rest conditions and pharmacologically induced vasodilatory conditions. In addition, we evaluate the use of FDG RBC PET imaging to characterize myocardial perfusion defects in a surgically induced myocardial infarction rat model. Finally, we show that FDG RBC PET imaging can detect differences in the degree of drug-induced vasodilation of the total LV myocardial vasculature between diabetic rats and normal rat controls.

## 2. Results

### 2.1. Detecting Changes in Total Rat Cerebrovascular Volume after Pharmacological Vasodilation

**Figure 1:**
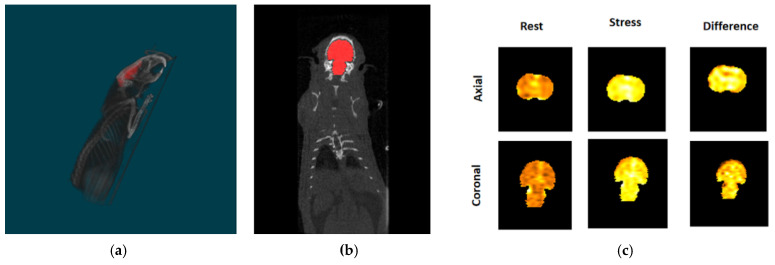
Sagittal (**a**) and coronal (**b**) PET/CT images of a representative rat brain segmentation. (**c**) FDG RBC tracer activity in the segmented rat brain under rest and pharmacological vasodilatory (“stress”) conditions, as well as the difference between the two after digital subtraction imaging (“Difference”). Total rats = 6.

**Figure 2:**
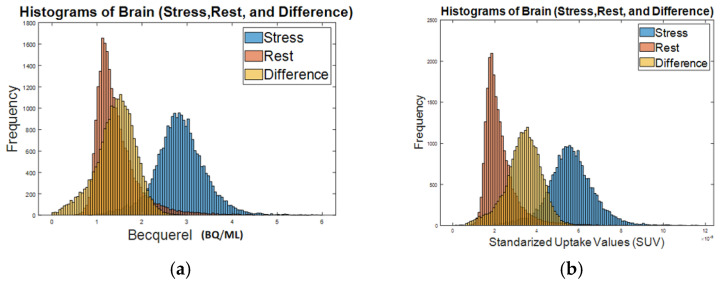
Histograms of FDG RBC tracer activity in a representative rat brain (pharmacological vasodilatory “stress”, rest, and the difference between stress and rest): (**a**) intensity activity (Bq/mL) in the brain; (**b**) standardized uptake value (SUV) in the brain.

We have previously shown that FDG-labeled human red blood cells (RBCs) can be used to visualize the total body vasculature of immunocompromised mice with a small animal microPET/CT scanner [28]. Similar to results shown in our prior publication, we found that after intravenous injection of FDG-labeled rat RBCs, there is diffuse, intense tracer activity throughout the rat brain volume on PET imaging (Figure 1). To determine the feasibility of using FDG RBC PET imaging to characterize the total in vivo vascular volume of the rat brain, we imaged the rat brain before and after administration of the pharmacological cerebral vasodilator acetazolamide (Figure 2). After administration of acetazolamide, there is a significant vasodilator-induced increase in the total cerebrovascular volume of 72.2 ± 14.7% (*n* = 6). The difference in the rat cerebrovascular volume between vasodilatory and rest conditions was statistically significant (two-tailed Wilcoxon signed rank test: U = 0 ≤ 2, α = 0.05, *n* = 6).

### 2.2. Detecting Changes in Rat LV Myocardial Volume after Pharmacological Vasodilation

**Figure 3:**
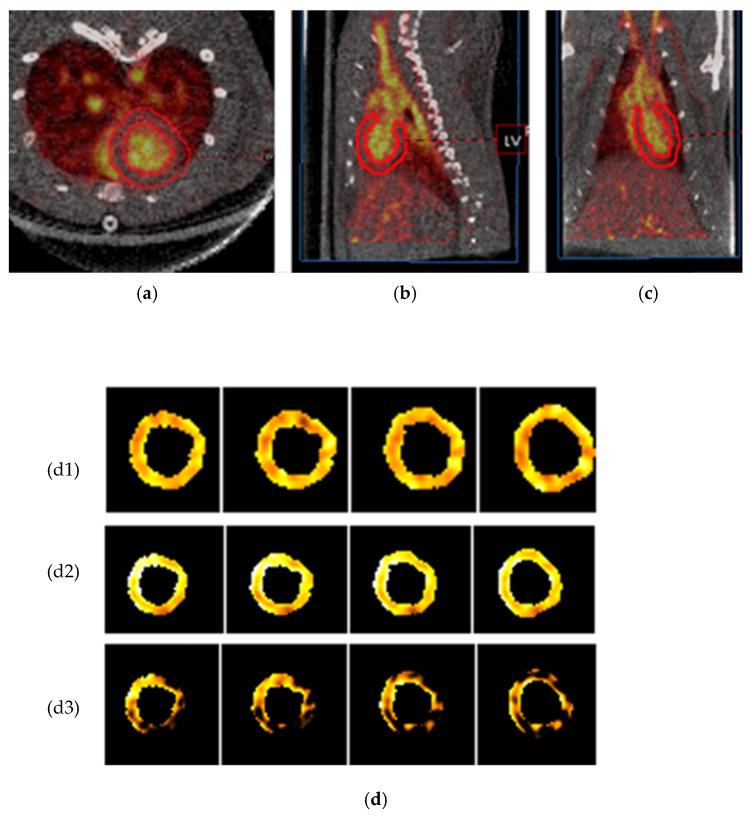
Volume segmentation of the left ventricular (LV) myocardium of a representative rat on FDG RBC PET/CT images. (**a**–**c**) Axial, coronal, and sagittal (respectively) segmentation of normal rat myocardium. Manual segmentation of the left ventricle is depicted by a red outline. (**d**) Four consecutive cardiac short axis views of a rat left ventricular wall under rest conditions (**d1**), vasodilatory stress conditions (**d2**), and the net activity difference between stress and rest conditions after digital subtraction imaging (**d3**). Total rats = 5.

**Figure 4:**
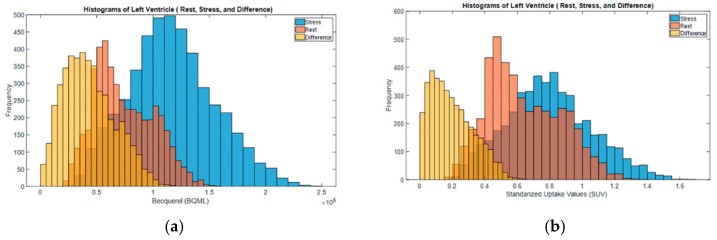
Histograms of FDG RBC tracer activity in the LV myocardial wall of a representative rat (Stress, after-stress = “rest” and the net difference). (**a**) Tracer activity (Bq/mL) in the LV myocardium. (**b**) Standardized uptake value (SUV) in the LV myocardium.

To further validate the utility of FDG RBC PET imaging to characterize organ vascularity, we showed that FDG RBC tracer activity can be directly visualized within the wall of the left ventricle (LV) in rats after manual image segmentation of the LV myocardium from the chamber blood pool activity on electrocardiogram (ECG)-gated PET images (Figure 3). In addition, we imaged the total LV intramyocardial vascular volume in rats under pharmacological stress conditions after injection of the coronary artery vasodilator regadenoson. Immediately after stress imaging was completed, the vasodilatory effects of regadenoson were pharmacologically reversed by administering a known antagonist, aminophylline, and the rat heart was imaged again (Figure 4). We were able to detect a relative pharmacologically induced difference in LV intramyocardial vessel volume with a mean volume difference of 52.3 ± 11.3% (Bq/mL) (*n* = 5). The volume difference was statistically significant (two-tailed Wilcoxon signed rank test: U = 0 ≤ 2, α = 0.05, *n* = 5). All myocardial image analysis was performed on ECG-gated binned images of the rat heart in the diastolic phase.

### 2.3. Imaging LV Intramyocardial Vascularity with FDG RBC PET in a Rat Myocardial Infarction Model

**Figure 5:**
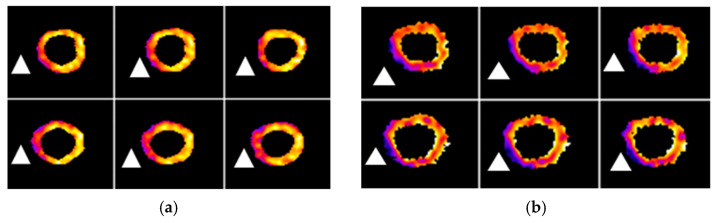
FDG PET and FDG RBC PET images of the LV myocardium (short axis view) from a representative myocardial infarction rat. (**a**) Metabolic FDG PET images. White triangles indicate the approximate location of decreased metabolism in the left ventricular wall. (**b**) FDG RBC PET images. White triangles indicate the approximate location of relative decreased myocardial perfusion/vascularity. Total rats = 6.

**Figure 6:**
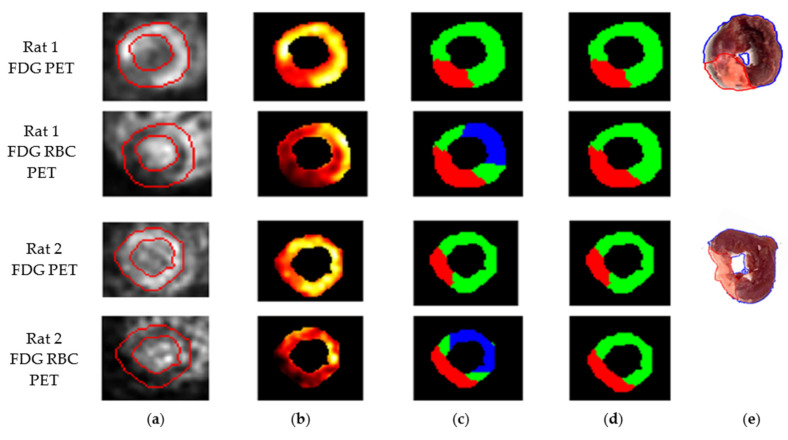
Two consecutive short axis views of the left ventricle from two representative myocardial infarction rats on metabolic FDG PET images and FDG RBC PET images. (**a**) LV myocardial segmentation on PET images. (**b**) Color heat map PET images of segmented LV. (**c**) Three-color stratified FDG RBC PET images (low = red, medium = green, high = blue) of tracer activity. (**d**) Two-color stratified PET image (red = infarct; green = viable). (**e**) TTC tissue viability staining of transverse slices of the LV myocardium from the same infarction rats. There is significant scar tissue and/or atrophy in the infarcted portion of the LV wall (red outline) of some of the rats compared to the uninjured myocardium (blue outline). Dark red tissue = viable myocardium. Tan tissue = infarcted myocardium. Total rats = 6.

We then sought to determine if FDG RBC PET imaging could detect and approximate the location of any myocardial perfusion defects in rats after surgical ligation of the left coronary artery. We also imaged the same rats after intravenous administration of pure FDG to identify any infarction-induced defects in myocardial FDG metabolism. The rats were sacrificed, the rat hearts were removed, and tissue viability staining was also performed. A representative short axis slice image of the left ventricle of each rat was then evaluated for infarct size based on each method. We found that the myocardial perfusion defect on FDG RBC PET images appeared to correlate in terms of approximate location with both the metabolic myocardial defect on intravenous FDG PET images as well as the infarct location on ex vivo myocardial tissue staining in these rats, when accounting for variability in myocardial infarct size between rats (Figure 5 and Figure 6). In some rats, the margins of the perfusion defect appeared to over-estimate the infarct size as measured on metabolic FDG images or with tissue staining, possibly reflecting areas of relatively decreased perfusion in otherwise viable peri-infarct myocardium (Table 1). The mean percent LV infarct size calculated from the representative slice image for each method was 26.9 ± 0.8% for FDG PET, 33.9 ± 1.6% for FDG RBC PET, and 26.0 ± 1.5% for TTC staining (mean ± S.E.) (Table 1). Further arbitrary segmentation of myocardial perfusion levels on imaging into a low, medium, or high range of tracer activity did not consistently improve visual interpretation of perfusion imaging in these rats, likely in part reflecting technical limitations in the tracer activity acquisition/resolution of our microPET/CT scanner.

### 2.4. Detecting Differences in Pharmacologically Induced Vasodilation in the Total Rat LV Intramyocardial Vasculature between Normal and Diabetic Rats

Diabetes is a prevalent disease in many countries associated with injury to both the macrovasculature and microvasculature of various organs, including the heart, brain, kidneys, and skeletal muscles [29]. Diabetic rats were created by intravenous injection of the drug streptozotocin, an antibiotic that is used to induce pancreatic islet β-cell destruction in rats [30]. Blood glucose was monitored weekly after the injection. Diabetic rats routinely showed blood glucose levels >400 mg/dL after a few weeks as well as loss of body weight, indicative of progressive diabetic pathophysiology. The mean blood glucose measurement (mg/dL) of the diabetic rats at the time of FDG RBC PET imaging was 394.0 ± 38.7 compared to 80.8 ± 12.7 for control rats (mean ± S.E.). The mean body weight (g) of the diabetic rats at that time was 306.8 ± 17.6 compared to 419.2 ± 8.3 for normal controls. Stress and rest myocardial PET imaging of diabetic and normal age-matched control rats was performed after injection of FDG-labeled RBCs. The diabetic rats were found to have a significantly lower degree of regadenoson-related LV myocardial vasodilation when compared to the control group. The mean percentage increase in LV intra-myocardial vascular volume after regadenoson injection in the diabetic rats was 18.8 ± 2.0% compared to 35.1 ± 1.9% in the control group (mean ± S.E.) (Table 2 and Table 3). The impairment in vasodilatory response in the diabetic group was statistically significant (two-tailed Wilcoxon rank-sum test: U = 0 ≤ 2, *p* < 0.05; diabetic rats, *n* = 5; normal controls, *n* = 5).

## 3. Discussion

In this work, we show that FDG RBC PET imaging can detect differences in the total vascular volume of the LV myocardial wall and whole brain of normal rats after pharmacologically induced vasodilation within these organs. In a surgical myocardial infarction rat model, we are also able to spatially correlate the approximate location of myocardial perfusion defects with both the myocardial abnormalities identified with metabolic FDG PET imaging and areas of infarcted myocardium determined by viability tissue staining. We further show in a diabetic rat model that FDG RBC PET imaging can detect impairments in drug-induced coronary vasodilation in the LV intramyocardial vascular bed compared to normal rats. As the microvasculature represents the vast majority of the total vessel volume of major organs, such as the heart (≥90% of the total intra-myocardial vasculature) [31], FDG RBC PET imaging offers the potential for non-invasive detection and serial assessment of the small vessel pathology in whole organs or large body parts seen in diabetic vasculopathy and other diseases.

Unlike other previously explored PET radiotracers, FDG RBC PET imaging offers the unique advantage of utilizing the relatively affordable and readily accessible PET tracer FDG. As described in our previous work, the cell labeling technique can be achieved in a relatively short time period and utilizes a straightforward cell incubation and washing protocol [28]. The cell labeling technique is also similar in the degree of complexity of 111-indium-labeled white blood cell imaging [32].

There is progressive development and ongoing adoption of time-of-flight (TOF) capabilities in modern PET/CT and PET/MR scanners which offer improvements in signal-to-noise ratios, image quality, and patient throughput. Recent studies combining TOF PET imaging with traditional myocardial perfusion agents are being used to quantify myocardial blood flow and improve detection of tracer uptake in lesions of interest [33]. Estimation of tumor flow has also been performed by dynamic PET imaging of the first vascular pass of FDG; however, this estimation requires flow modeling with dynamic measures of arterial tracer concentration. It also requires accurate estimation of the presumed FDG extraction rate parameter by the target tissue and may be susceptible to mild underestimation of blood flow [34]. It remains to be seen whether first-pass dynamic FDG PET imaging or TOF PET imaging can reliably quantify the total vascular volume of an organ or tissue of interest. Unlike first pass FDG PET imaging or TOF PET imaging, FDG RBC PET imaging of the blood pool should allow for vascularity assessment of multiple organs or tissues of interest across different fields of view.

Quantitative measures of total organ vasculature may be possible with clinical PET/CT scanners using FDG RBC PET/CT, based on CT segmentation-based organ volume calculations normalized to measure in vivo FDG RBC concentrations within a large, segmented vessel (aorta, vena cava). Our current goals include exploring the potential of FDG RBC PET imaging to assess in vivo tumor vascularity response to anti-angiogenic drugs for our oncological patient population. FDG RBC PET imaging may also be useful for the translational researcher seeking to non-invasively assess the efficacy of promising pharmaceutical agents targeting other microvascular disease in both small animal models and human subjects.

## 4. Materials and Methods

All experimental procedures were approved by the University of South Florida (USF) Institutional Animal Care and Use Committee (IACUC). All experiments were performed in accordance with U.S.A. federal regulations and USF IACUC principles and procedures. All of the chemicals were obtained from Sigma-Aldrich, St. Louis, MO, USA, unless specified. A total of ten 4–8-week-old male Sprague-Dawley (SD) rats were used, including four healthy male rats and six myocardial infarction rats. The vendor-supplied myocardial infarction rats were created using a standardized surgical ligation technique of the proximal left coronary artery with an estimated ~35–45% LV infarct size (Envigo, Indianapolis, IN, USA). For drug-induced increases in vascular volume experiments, four 4–8-week-old SD rats were used for cerebrovascular imaging, and five 4–8-week-old SD rats were used for intra-myocardial vascular imaging. For the creation of a diabetic rat model, five 2–4-month-old SD male rats were intravenously injected once or twice with streptozotocin (40 mg/kg weight), and blood glucose was monitored weekly. Diabetic rats underwent myocardial microPET/CT imaging approximately 5 or more weeks after streptozotocin injection. Diabetic rats were compared to five normal age-matched control male SD rats.

### 4.1. Myocardial and Cerebral Vascular Value Measurement

The total myocardial and cerebral vascular tracer activity was measured in both rest and vasodilatory stress conditions with microPET/CT imaging. Stress condition myocardial PET imaging was performed after intravenous injection of the pharmaceutical coronary vasodilator regadenoson (25 μg/kg). Coronary artery vasodilation was then pharmacologically reversed by intravenous administration of aminophylline (40 mg/kg), and cardiac imaging was repeated. “Stress” condition cerebrovascular PET imaging was performed after intravenous injection of the pharmaceutical cerebral venous vasodilator acetazolamide (100 mg/kg). To segment the left ventricle (LV), the PET and CT images were loaded in Mirada Medical software (Mirada DBx 2.1.0, Denver, CO, USA) to align both image series together. CT images were used to identify the outer margins of the left ventricle, while PET images were used to define the boundaries that separate the left ventricular wall from the intraluminal blood chamber and other soft tissues. Using the built-in segmentation tools offered by Mirada, the left ventricular myocardium was manually delineated voxel by voxel in each short-axis slice (~25 slices, depending on the animal). The segmentation was further refined by adjusting the segmented LV boundaries in the coronal and sagittal views. Finally, any papillary muscles or surrounding soft tissue were excluded from the segmentation. Both normal SD rats and diabetic SD rats underwent stress and rest myocardial PET imaging. The same general approach was also used for the rat brain segmentation for normal rats. The histograms of the LV myocardial becquerel (Bq) activity and standardized uptake values (SUV) (stress, after-stress, and difference) were quantified.

### 4.2. FDG-Labeled RBC Preparation

Rat red blood cells were labeled with FDG using a previously published protocol [28], with minor differences, described below. About 500–1000 μL rat blood was collected in a heparin phlebotomy tube through the rat saphenous vein and stored in a 37 °C tissue culture incubator (Sanyo Scientific, New York, NY, USA) for 1–2 h to increase erythrocyte and plasma glucose depletion. After that, rat erythrocytes were centrifuged 1000× *g* for 10 min, and the plasma and buffer coat were gently aspirated. The remaining red blood cell pellets were gently resuspended in 4X volume of filter-sterilized “1X EDTA” solution (140 mM NaCl, 4 mM KCl, 2.5 mM ethylenediaminetetraacetic acid dipotassium salt dihydrate (K_2_EDTA dihydrate)), and centrifuged 1000× *g* for 10 min. The wash buffer was gently aspirated, and 100 μL 5X EDTA solution and 50 μL deionized water were then added to the 250 μL washed erythrocytes. Finally, 100 μL (37–74 MBq) USP grade 2-deoxy-2[18F]-fluoro-2-D-glucose (FDG) (Cardinal Health, Tampa Fl, USA) was added to a final volume of 500 μL. Samples were incubated at 37 °C for 30 min, centrifuged 3 times and washed 3 times with 12X volume of 1x EDTA/5 mM glucose solution. First, 18F-FDG-labeled RBC PET/CT imaging was performed. For the myocardial infarction rats, myocardial PET imaging was then performed in the same rat after intravenous injection of pure 18F-FDG within the following week. After image processing, the rat was sacrificed. The rat heart was excised intact, saline flushed, and then placed in a −20 °C freezer for 30 min. The semi-frozen rat heart was cut transversely into 2 mm thick sections within a 3D printed rat heart mold for uniform transverse sectioning. The heart tissue was then stained in 1% 2,3,5-triphenyl tetrazolium chloride (TTC) buffer solution at 37 °C for 20–30 min [35]. Stained myocardial slices were subsequently treated with 10% formalin for 20–30 min [36]. Digital images of the formalin-treated heart slices were obtained.

### 4.3. Small Animal PET/CT Imaging

A more detailed description of our imaging protocol has been previously published [30]. The rat was anesthetized via a nosecone manifold under 2–4% inhalational isoflurane. A tail vein micro-catheter was inserted into one of the dilated rat tail veins after warming the rat tail. The rat was then secured onto the micro-PET/CT scanner bed under anesthesia. Then, 250–500 µL of FDG-labeled RBC suspension (3.7 × 10^7^–1.01 × 10^8^ Bq) was injected slowly through the tail vein microcatheter, after which CT calibration imaging was performed. Electrocardiogram (ECG) leads were placed on two front limbs and one hind limb of the rat (ground lead on a rear leg) for ECG-gated PET imaging. The signals detected by these electrodes were recorded during the 20 min time period using a BioVet^®^ physiological monitoring and heating system (m2m Imaging, Richmond Heights, OH, USA). The threshold for TTL cardiac gating signals was set in a rising mode of R-wave peak. PET list-mode data were reconstructed using 3D-OSEM (ordered-subset expectation maximization) iterative algorithm with four iterations and eight subsets, with a final image volume of 256 × 256 × 256 voxels. Effective voxel dimensions were set at 1.4 mm × 1.4 mm × 1.4 mm. For each animal, there are three data sets: standard three-dimensional (3D) PET reconstruction, resulting in a motion–time average 3D PET image; dynamic 3D PET reconstruction with 30 frames; and the phase-based four-dimensional (regular 3D plus time, 4D) PET cardiac reconstruction, with four cardiac gate binning.

### 4.4. PET Image Analysis

The whole-body PET images of the rats were acquired using Siemens Inveon Workstation Software (Siemens Medical Inc., Knoxville, TN, USA). For 3D PET and 4D PET data sets, multiple volumes of interest (VOI) were selected based on corresponding CT as needed. Voxel activities were represented in standardized uptake values (SUVs). Dynamic activity curves for VOIs were obtained using the dynamic 3D PET data set for each animal. The 4D PET data were used to define cardiac function. ECG-gated binning of images of the heart in diastolic phase was performed. Heart segmentation on the CT images was based on anatomical features, and the segmented volume was transferred for image-co-registration (cardiac PET VOI). After segmentation of the left ventricular myocardium, the segmentation was then used to obtain the myocardial tracer activity inside the LV muscle. The activity values were converted to SUV units using the following formula: SUV (g/mL) = voxel value × (C × (weight (kg))/(dose (Bq))) × 1000 (g/kg), where C is the correction factor for 18-fluorine tracer decay. Images were represented as maximum intensity projection (MIP) reconstructions of the source data.

The difference in tracer activity within the rat LV myocardium between the vasodilatory “stress” state and the “rest” state were obtained by co-registration of the segmented rat brain or rat left ventricular (LV) myocardium. The difference in activity of the co-registered images was calculated on a pixelwise basis. Pixel differences that yielded negative values are thought to largely reflect volume averaging artifacts related to the abutting high tracer activity of the blood within the LV chamber, and were thus set to zero.

### 4.5. Myocardial Infarction Size Measurement

The rat heart slices were stained with triphenyl tetrazolium chloride (TTC) to identify the myocardial infarct region. The digitized images were analyzed with ImageJ software (National Institutes of Health, Bethesda, MD, USA) using the “color threshold” mode to manually delineate and measure infarcted vs. viable myocardial tissue. Left ventricular short axis images were obtained from both FDG images and FDG RBC images and then compared to TTC stained images.

Regions of decreased FDG RBC tracer activity on FDG RBC PET images and decreased FDG metabolism on FDG PET images were presumed to correspond to infarcted myocardium and delineated from areas of uninjured myocardium. The infarct size was estimated as a percentage of the left ventricular myocardial cross-sectional area on a given short axis slice. In addition, the distribution of activity inside the myocardial muscle was also arbitrarily further divided into three subregions of tracer activity: “low,” “medium,” and “high” activity, by first using the OTSU thresholding algorithm [37]. The OTSU algorithm evaluates the activity distribution and divides it into three sub-divisions based on the shape of the activity histogram. The three regions defined by OTSU represent low, medium, and high perfusion subregions inside the myocardium. The medium and high perfused subregions were considered viable myocardium and excluded from the final infarct estimate. Further fine adjustment of the boundaries of the three subregions was performed manually with advice from an experienced radiologist (JWC).

The relative infarct percentage was defined as infarct percentage = (area of the infarcted muscle (low))/(area of all muscle (low + medium + high)). The other two regions, high and medium, were considered uninjured heart muscle.

## 5. Conclusions

We present data that FDG-labeled erythrocytes can be used to characterize pharmacologic induced changes in the total vascularity of the rat myocardium and rat brain with PET/CT imaging. We also present data that FDG-labeled erythrocyte PET (FDG RBC PET) imaging can detect abnormalities in the left ventricular myocardium of both a surgical myocardial infarction rat model and a diabetic rat model. FDG RBC PET imaging may thus be useful for non-invasively assessing microvascular disease in various clinical settings. It may also be useful for evaluating potential drug candidates targeting microvascular disease.

## 6. Patents

A provisional patent application regarding the use of FDG RBC PET imaging for imaging microvascular disease was filed by the H. Lee Moffitt Cancer Center & Research Institute, Tampa, Florida, U.S.A., in July 2021.

## Figures and Tables

**Table 1:** Results of mean infarction size measurement based on FDG RBC PET images, FDG PET images, and TTC pathology staining.

Rat	FDG%	RBC%	TTC%
1	24.38%	37.19%	25.97%
2	29.19%	29.49%	26.28%
3	25.46%	32.85%	23.58%
4	29.26%	29.81%	21.35%
5	27.46%	35.39%	31.90%
6	25.62%	38.64%	27.04%
Mean ± S.E.	26.93 ± 0.83%	33.89 ± 1.56%	26.02 ± 1.45%

**Table 2:** Tracer activity (Bq/mL) in the segmented LV intra-myocardial vascular volume in stress and rest conditions and relative percentage stress-related increase in LV intra-myocardial vascular volume (“stress–rest difference”) after regadenoson injection in the control rats. Stress–rest difference is calculated on a pixelwise level, as detailed in the Materials and Methods section.

Control Rat	LV Stress Activity	LV Rest Activity	Stress–Rest Difference	Increased Ratio
1	2496.72	2008.44	635.04	31.62
2	14,927.50	9340.60	6061.95	32.49
3	12,271.33	9568.55	3108.66	41.00
4	20,315.93	14,905.81	5483.7	36.79
5	46,501.94	34,927.14	11,760.07	33.70
Mean (± S.E.)				35.12 ± 1.91

**Table 3:** Tracer activity (Bq/mL) in the segmented LV intra-myocardial vascular volume in stress and rest conditions and relative percentage stress-related increase in LV intra-myocardial vascular volume (“stress–rest difference”) after regadenoson injection in the diabetic rats. Stress–rest difference is calculated on a pixelwise level, as detailed in the Materials and Methods section.

Diabetic Rat	LV Stress Activity	LV Rest Activity	Stress–Rest Difference	Increased Ratio
1	3386.6	3042.22	410.54	13.40
2	14,225.28	14,026.88	2195.59	15.56
3	18,781.97	15,579.75	3796.07	24.37
4	6124.33	5829.02	1084.05	18.59
5	7063.27	5778.81	1284.46	22.20
Mean (±S.E.)				18.82 ± 2.03

## Data Availability

The data presented in this study are available on request from the corresponding author. The data are not publicly available due to the filing of a provisional patent application by the H. Lee Moffitt Cancer Center and Research Institute covering this body of research.
